# Supplementary material for: Content-rich biological network constructed by mining PubMed abstracts
Source: BMC Bioinformatics. 2004 Oct 8;5:147. doi: 10.1186/1471-2105-5-147 (PMC528731; doi:10.1186/1471-2105-5-147)
Supplement: Additional File 2 — The original results of the above study (non-essential files are deleted to keep the file size under the limit set by BMC bioinformatics). [file 1471-2105-5-147-S2.bz2 › chilibotAdditionalFile2/dip50/237ID9211894E766/html/AR_JUN.html]

 


 **AR** and **JUN** 
  
Found 81 abstracts in PubMed, retrieved 50.  
 

 What does Google say? 
 PDF only 
| .edu only 

---

**Interactive relationship** (e.g. stimulation, inhibition, etc)

**Inhibitory relationship**- A transient transfection found that forced expression of c  **jun**  inhibited  **AR**  transactivation activity.  Ref: 11470750 Carcinogenesis, 2001
- Conversely, when activated, AP 1  [ **JUN** ]  can block  **AR**  activity.  Ref: 9822653 J Biol Chem, 1998
- In addition to its ability to activate transcription from androgen response elements,  **AR**  can inhibit activator protein 1  [ **JUN** ]  AP 1  [ **JUN** ]  activity, composed of  **Jun**  and Fos oncoproteins, in a ligand dependent manner.  Ref: 9822653 J Biol Chem, 1998
- These results suggest that AhR can stimulate AP 1  [ **JUN** ]  expression resulting in inhibition of the binding of  **AR**  to ARE in the transcription regulatory region of target genes such as PSA.  Ref: 12799773 Arch Toxicol, 2003
- Inhibition of  **AR**  also attenuated the increase in p38 mitogen activated protein kinase and c  **Jun**  N terminal kinase phosphorylation.  Ref: 12490536 FASEB J, 2003
**Neutral relationship**- CREB binding protein is a coactivator for the androgen receptor  [ **AR** ]  and mediates cross talk with AP 1  [ **JUN** ] .  Ref: 9822653 J Biol Chem, 1998
- All mutants efficiently interfered with AP 1  [ **JUN** ]  activity, indicating that ligand dependent activation of transcription and interference with AP 1  [ **JUN** ]  activity are separable functions in  **AR** .  Ref: 11043576 Mol Endocrinol, 2000
- Collectively, these data indicate that several segments of the androgen receptor  [ **AR** ]  are involved in cross talk with the AP 1  [ **JUN** ]  pathway.  Ref: 9886845 Endocrinology, 1999
- c  **Jun**  potentiates the functional interaction between the amino and carboxyl termini of the androgen receptor  [ **AR** ] .  Ref: 11577103 J Biol Chem, 2001
- In neonatal cardiomyocytes, activation of the G coupled alpha adrenergic receptor  **AR**  induces hypertrophy by activating mitogen activated protein kinases, including c  **Jun**  NH terminal kinase.  Ref: 12433842 Circ Res, 2002
- One such factor is c  **Jun** , which has been shown to have a selective function of mediating androgen receptor  [ **AR** ]  dependent transactivation.  Ref: 9867253 Endocrine, 1998
- Transcriptional repression functions of the wild type and chimeric receptors were measured using an activator protein 1  [ **JUN** ]  AP 1  [ **JUN** ]  transrepression assay and identified the GR HBD as a more potent transrepressor of AP 1  [ **JUN** ]  transcriptional activation than the  **AR**  HBD.  Ref: 11572317 Endocrine, 2001
- Inappropriate interactions between c  **Jun**  and androgen receptors containing pathological length glutamine repeats may therefore be part of the pathogenic process in SBMA  [ **AR** ] .  Ref: 10643885 Neurosci Lett, 1999
- The  **AR**  acetylation site point mutants showed wild type trans repression of NF kappa B, AP 1  [ **JUN** ] , and Sp1 activity.  Ref: 11971970 Mol Cell Biol, 2002
- In addition to this genomic action, the  **AR**  also interacts with other signaling pathways through protein protein interaction, for example with AP 1  [ **JUN** ]  or Ets transcription factors.  Ref: 10325498 Eur Urol, 1999
- In addition, liganded  **AR**  can interfere with the activity of other transcription factors, such as activator protein 1  [ **JUN** ]  and nuclear factor kappaB, for which DNA binding by  **AR**  is not necessary.  Ref: 11429409 J Biol Chem, 2001
- c Fos dimerization with c  **Jun**  represses c  **Jun**  enhancement of androgen receptor  [ **AR** ]  transactivation.  Ref: 9867253 Endocrine, 1998
- Here we show that beta  **AR**  stimulation activates p38 kinase, c  **jun**  N terminal kinases JNKs, and extracellular signal regulated kinase ERK1 2 in ARVMs.  Ref: 10770956 J Biol Chem, 2000
- Furthermore, with increasing Ro 31 8220, beta 2  **AR**  promoter reporter activity correlated closely with both NF kappa B and AP 1  [ **JUN** ]  activities r > 0.89 for both.  Ref: 11597920 Am J Physiol Lung Cell Mol Physiol, 2001

**Non-interactive relationship** (e.g. studied together, co-existance, homology, etc.)

- The androgen receptor  [ **AR** ]  and c  **Jun**  are known to interact to modulate each others transcriptional activities.  Ref: 10643885 Neurosci Lett, 1999
- The remaining 3.5 kb upstream flanking sequence contains no apparent TATA box but has several sequences that resemble binding sequences for transcription factors such as androgen receptor  [ **AR** ]   **AR** , AP1  [ **JUN** ] , AP2, AP4, Sp1, MyoD, Myc, and CArG.  Ref: 11162575 Biochem Biophys Res Commun, 2001
- We examined the 500 bp upstream of exon 1 in all three H. burtoni genes and identified putative binding sites for glucocorticoid receptor, androgen receptor  [ **AR** ] , and progesterone receptor, as well as the transcription factors Ap 1  [ **JUN** ]  and Sp 1.  Ref: 9748399 Gen Comp Endocrinol, 1998
- c  **Jun**  targets amino terminus of androgen receptor  [ **AR** ]  in regulating androgen responsive transcription.  Ref: 11051047 Endocrine, 2000
- NE also activated luciferase reporter constructs for seven different transcription factors AP1  [ **JUN** ] , SRE, CRE, NFkappaB, NFAT, Stat, GAS following transfection into alpha1A  **AR**  expressing PC12 cells.  Ref: 11358336 Life Sci, 2001
- Finally, the selective NF kappa B inhibitor MG 132 dose dependently reduced NF kappa B binding and beta 2  **AR**  promoter activity but increased AP 1  [ **JUN** ]  binding.  Ref: 11597920 Am J Physiol Lung Cell Mol Physiol, 2001
- We report that, identically to 17beta estradiol or dihydrotestosterone, but differently from raloxifene, estren alters the activity of Elk 1, CCAAT enhancer binding protein beta C EBPbeta, and cyclic adenosine monophosphate response element binding protein CREB, or c  **Jun**  c Fos by an extranuclear action of the ER or  **AR** , resulting in activation of the Src Shc ERK pathway or downregulation of JNK, respectively.  Ref: 12782668 J Clin Invest, 2003
- In our previous study, we found a new mixed formula of Chinese herbs containing Shin yi san Xiao qing long tang Xiang sha liu  **jun**  zi tang 9 3 3 g divided in three doses day was beneficial to the patients with perennial allergic rhinitis  **AR**  via complicated immunomodulatory effects on both mononuclear cells MNC and polymorphonuclear neutrophils PMN.  Ref: 12469937 Int Immunopharmacol, 2002
- The alpha 2  **AR**  agonists, clonidine and UK14304 also had no effect on basal IEG levels, while blockade of alpha 2 ARs by methoxyidazoxan significantly increased NGFI A and c fos expression, but decreased c  **jun**  mRNA levels.  Ref: 11072104 Brain Res Mol Brain Res, 2000
- Agonist activation of the alpha  **AR**  resulted in increased p38 kinase activity, but not c  **Jun**  NH terminal kinase activity.  Ref: 11752101 J Pharmacol Exp Ther, 2002
- Our results show that curcumin down regulates transactivation and expression of  **AR** , activator protein 1  [ **JUN** ] , nuclear factor kappaB, and CREB binding protein.  Ref: 12239622 Int J Oncol, 2002
- Here we show that the size of this polyglutamine tract influences both c  **Jun**  regulation of androgen receptor  [ **AR** ]  mediated transcription and androgen receptor  [ **AR** ]  regulation of c  **Jun**  activity.  Ref: 10643885 Neurosci Lett, 1999
- We investigated the interferences of the normal or mutated androgen receptor  [ **AR** ]  with the activator protein 1  [ **JUN** ]  AP 1  [ **JUN** ]  by assessing their effects on transcriptional activity in CV 1 cells.  Ref: 9886845 Endocrinology, 1999
- After nerve injury, double labelling with Fluoro Gold, a marker of retrograde transport from transected fibres, or by immunoreactivity for c  **jun**  protein, an indicator of injury and regeneration, suggested that many of the neurones expressing alpha2A  **AR**  IR were uninjured by the sciatic lesions.  Ref: 10050019 J Physiol, 1999
- AP1  [ **JUN** ]  function was repressed not only by wild type  **AR**  but also by the transactivating mutants A579T and R590Q as well as by the transcriptionally inactive mutants G551V and C562G.  Ref: 10385402 Endocrinology, 1999
- Although the AP 1  [ **JUN** ]  c  **Jun**  c Fos system and NcoR have been reported to interact with and repress the activity of some steroid receptors, c  **Jun** , c Fos, c  **Jun**  c Fos, nor NcoR function was consistently affected by the absence or presence of residues 628 646, implying that the  **AR**  hinge region exerts its silencing effects in a manner independent of these corepressors.  Ref: 11102454 J Biol Chem, 2001
- In PC12 cells stably transfected with the human alpha 1A  **AR** , norepinephrine NE strongly activated both extracellular signal regulated kinases ERKs and c  **jun**  NH2 terminal kinases JNK.  Ref: 9927621 Mol Pharmacol, 1999
- Since activator protein 1  [ **JUN** ]  AP 1  [ **JUN** ] , a heterodimer of c  **jun**  and c fos proteins, is known to inhibit binding of  **AR**  to ARE by protein protein interaction with  **AR** , the findings in the present study suggest a possible involvement of AP 1  [ **JUN** ]  in the antiandrogenic effects of PAHs acting as AhR agonists.  Ref: 12799773 Arch Toxicol, 2003
- Furthermore, recombinant PIASxalpha enhances Ubc9 mediated sumoylation of the androgen receptor  [ **AR** ]  and c  **Jun**  in vitro.  Ref: 12077349 Mol Cell Biol, 2002
- Polyglutamine repeat length influences human androgen receptor  [ **AR** ]  c  **Jun**  mediated transcription.  Ref: 10643885 Neurosci Lett, 1999
- The blockade of dephosphorylation by protein phosphatases identifies the protein phosphatases that modulate the AP 1  [ **JUN** ]  androgen receptor  [ **AR** ]  cross talk.  Ref: 9886845 Endocrinology, 1999
- Using engineered or naturally occurring androgen receptor  [ **AR** ]  mutants that are responsible for complete or partial androgen insensitivity syndromes, we defined the subregions involved in the cross talk of the androgen receptor  [ **AR** ]  with the AP 1  [ **JUN** ]  factors.  Ref: 9886845 Endocrinology, 1999
- On the other hand, repression of  **AR**  activity by treatment of LNCaP cells with an activator of AP 1  [ **JUN** ]  was largely relieved when CBP was ectopically expressed.  Ref: 9822653 J Biol Chem, 1998
- Exogenous c  **jun**  enhanced transcriptional activation by the  **AR**  in a ligand dependent manner, but had no effect in the absence of DHT.  Ref: 9780230 Eur J Biochem, 1998
- the transcriptional interference between  **AR**  and AP 1  [ **JUN** ]  is the result of competition for limiting amounts of CBP in the cell.  Ref: 9822653 J Biol Chem, 1998
- Furthermore, a Lys to Ala substitution in codon 563 of the first ZF switched  **AR**  into a ligand dependent activator at AP1  [ **JUN** ]  sites but maintained the ability to repress NF kappaB RelA function.  Ref: 10385402 Endocrinology, 1999
- Transcriptional interferences between normal or mutant androgen receptors and the activator protein 1  [ **JUN** ]  dissection of the androgen receptor  [ **AR** ]  functional domains.  Ref: 9886845 Endocrinology, 1999
- Thus, this study found that the inhibitory effects of omega 3 polyunsaturated fatty acids on  **AR**  mediated actions are due, at least in part, to an increase in c  **jun**  protein.  Ref: 11470750 Carcinogenesis, 2001
- ... inhibition of c Fos, c  **Jun** , HB EGF,  **AR** , and VEGF mRNA by this compound was incomplete 41 65%.  Ref: 12016209 J Biol Chem, 2002
